# New Perspectives on the Aberrant Alveolar Repair of Idiopathic Pulmonary Fibrosis

**DOI:** 10.3389/fcell.2020.580026

**Published:** 2020-09-30

**Authors:** Zhao Ni Wang, Xiao Xiao Tang

**Affiliations:** State Key Laboratory of Respiratory Disease, National Clinical Research Center for Respiratory Disease, Guangzhou Institute of Respiratory Health, The First Affiliated Hospital of Guangzhou Medical University, Guangzhou, China

**Keywords:** idiopathic pulmonary fibrosis, alveolar regeneration, lung epithelial progenitor/stem cells, lung injury, microenvironment

## Abstract

Idiopathic pulmonary fibrosis (IPF) is a chronic lung disease of unknown etiology and high mortality. Current therapeutic strategies have limited efficacy and the prognosis remains poor. Based on the histological observations of IPF lung tissues and experimental studies using lung fibrosis animal models, it is gradually accepted that impaired epithelial regeneration after lung injury is a critical mechanism underlying the pathogenesis of pulmonary fibrosis. The central role of AEC2 in this process has been well-elucidated, while the contribution of other lung progenitor/stem cells is less discussed. Recently, increasing studies have identified several non-AEC2 epithelial progenitor/stem cells with great plasticity to transform into mature AECs and reconstitute alveolar epithelium after lung injury. However, why these cells do not function as alternate stem cells to regenerate alveolar epithelium in IPF is still unknown. In this review, we discuss the contribution of lung epithelial progenitor/stem cells in the aberrant alveolar regeneration, and provide a novel perspective on the mechanism of IPF pathogenesis, in which non-AEC2 progenitors may play an essential role.

## Introduction

Idiopathic pulmonary fibrosis (IPF) is the most common form of idiopathic interstitial pneumonia, with annual incidence of 1.7–27.1 cases per 100,000 persons (Raghu et al., 2006, 2016; Natsuizaka et al., 2014; Gjonbrataj et al., 2015; Harari et al., 2016, 2020; Hopkins et al., 2016; Strongman et al., 2018). IPF is a rapid, progressive and fatal disease, leading to irreversible pulmonary dysfunction and a median survival of only 2–5 years from diagnosis (Raghu et al., 2011; Caminati et al., 2015; Strongman et al., 2018). At present, there are only two FDA-approved drugs for patients with IPF — nintedanib and pirfenidone. Both of them show an effect on improving lung function (Noble et al., 2011; King et al., 2014; Richeldi et al., 2014), but fail to reverse lung fibrosis or bring further survival benefit. Lung transplantation may be the only way to improve both life quality and survival rate. However, its application is greatly hampered by surgery complexity, limited supply of donor organs and expensive cost (George et al., 2019).

The pathogenesis in IPF is not fully understood. Historically, IPF was thought to be a chronic inflammatory disorder, since it appeared to be UIP (Raghu et al., 2011), with infiltration of alveolar macrophages or other immune cells (Misharin et al., 2017). However, increasing evidences are against this concept (Katzenstein and Myers, 1998; Idiopathic Pulmonary Fibrosis Clinical Research Network et al., 2012). Now a more acceptable theory regarding the disease pathogenesis believes that IPF results from the vicious cycle of “alveolar epithelial injury – abnormal repair” (Gross and Hunninghake, 2001).

## Aec2 Dysfunction in IPF

Type II alveolar epithelial cells (AEC2) serve as the prime progenitor/stem cells for alveolar regeneration during tissue repair and homeostasis (Aso et al., 1976; Selman and Pardo, 2006; Barkauskas et al., 2013). Depletion and dysfunction of AEC2 play a central role in the pathogenesis of lung fibrosis. The percentage of AEC2 in total alveolar epithelial cells declines dramatically in IPF patients (Xu et al., 2016). The death of AEC2 is associated with activation of apoptotic pathway (Maeyama et al., 2001), increased endoplasmic reticulum (ER) stress (Korfei et al., 2008) and mitochondrial dysfunction (Bueno et al., 2015). In experimental animals, targeted ablation of AEC2 is sufficient to induce lung fibrosis (Sisson et al., 2010). Furthermore, accelerated epithelial cell senescence, evidenced by the expression of p21 and senescence-associated β-galactosidase (SA β-gal) in lung epithelial cells of IPF patients, may be another major contributor to lung fibrosis (Minagawa et al., 2011; Tian et al., 2019). Recent studies have successfully established several animal models of progressive lung fibrosis via inducing epithelial cell senescence (Naikawadi et al., 2016; Yao et al., 2019) or impairing renewal ability in AEC2 (Wu et al., 2020). AEC2 dysfunction is multifactored as it can result from intrinsic genetic mutations and/or external stimuli. Genomic analysis has revealed several genetic variants correlated with lung fibrosis, such as surfactant protein C (*SPC*) (Thomas et al., 2002), *MUC5B* (Seibold et al., 2011), telomerase reverse transcriptase (TERT) (Mushiroda et al., 2008; Moore et al., 2019), telomerase RNA component (TERC) (Moore et al., 2019) and regulator of telomere elongation helicase 1 (RTEL1) (Moore et al., 2019). The exact function of *SPC* and *MUC5B* in lung fibrosis is still under investigation, while the latter three genes have been found to be linked to premature senescence of AEC2. The secretome of senescent epithelial cells, including IL-1β, IL-6 and IL-8, promotes the differentiation of fibroblasts into myofibroblasts and their resistance to apoptosis, and thus leads to the progression of lung fibrosis (Minagawa et al., 2011; Chen et al., 2019; Tian et al., 2019). Environmental exposures like smoking and viral infection are also risk factors for IPF (Baumgartner et al., 1997; Sheng et al., 2020), as they may impair AEC2 function via elevating ER stress and inducing DNA damage response.

The hyperplasia of AEC2 and loss of type I alveolar epithelial cells (AEC1) also implicate that the transdifferentiation of AEC2 into AEC1 is halted. The AEC2-to-AEC1 differentiation is at least partially regulated by TGF-β and BMP pathways (Zhao et al., 2013; Dituri et al., 2019). These two signaling proteins function in contrary ways and inhibit each other. TGF-β promotes the loss of AEC2 phenotype and induces the expression of AEC1 markers, while BMP inhibits the differentiation of AEC2 and enhances their proliferation. *In vitro* study showed that TGF-β was activated naturally during the differentiation of AEC2 into AEC1, while BMP and its downstream factors were suppressed (Zhao et al., 2013). Choi et al. (2020) demonstrated that IL-1β was also required to trigger the reprogramming of AEC2 during alveolar regeneration, but sustained IL-1β treatment hindered the generation of mature AEC1 via inducing expression of *Hif1a* and other glycolysis pathway genes. Thus, TGF-β/BMPs imbalance, or chronic IL-1β-induced inflammation may lead to epithelial cell failure and lung fibrosis (Gu et al., 2014; Lasithiotaki et al., 2016).

Due to its insidious onset, IPF is thought to result from repetitive stimuli-induced mild injuries to lung epithelium (Selman et al., 2001), leading to ablation of surface pneumocytes and initiating wound healing response, such as the accumulation of lung fibroblasts and extracellular matrix. When alveolar stem cells like AEC2 fail to regenerate normal alveolar epithelia and produce sufficient surfactant proteins, the alveolar epithelium will confront secondary injury from the increased mechanical tension, which aggravates epithelial cell depletion and triggers the profibrotic TGF-β signaling in AEC2 (Wu et al., 2020). This activated pathway can promote fibrogenesis (Cabrera-Benítez et al., 2012; Wu et al., 2020), as well as suppress the transdifferentiation of AEC2 into AEC1 (Riemondy et al., 2019), disturbing the lung regeneration process, thus a vicious cycle forms.

However, although the role of AEC2 dysfunction in IPF has been well-delineated, something remains elusive. In animal models of lung fibrosis, depletion of mouse AEC2 does not always result in a sustained lung fibrosis. For example, conditionally ablation of AEC2 in bleomycin-injured mice did not bring about persistent lung fibrosis (Salwig et al., 2019). This indicates that other than AEC2 dysfunction, some additional mechanisms may also be defective in the progression of IPF.

### Potential Role of Other Epithelial Progenitor/Stem Cells in IPF

According to the current theory, the resolution of lung fibrosis bases on functional restoration of alveolar epithelia. When AEC2 are depleted or lose their regeneration capability, progression of lung fibrosis may hinge on whether there are other alternate lung progenitor/stem cells to reconstitute the ablated alveolar epithelia. Lineage tracing studies in mice have identified several types of non-AEC2 epithelial progenitor/stem cells with the capability to restore alveolar epithelia via differentiating into AEC1 and/or AEC2 (Table 1).

**TABLE 1:** Progenitor/stem cells involved in the alveolar regeneration after injury.

**Progenitor/stem cells**	**Selective markers**	**Localization**	**Daughter cells**	**Reported in**	**Contributions to alveolar repair**	**References**
AEC2	SPC^+^/HTII-280^+^	Alveoli	AEC2; AEC1	Human; bleomycin-injured mice	AEC2 can self-renew and give rise to AEC1 during normal condition or under injury.	Aso et al., 1976; Barkauskas et al., 2013
AEC1	Hopx^+^Igfbp2^–^	Alveoli	AEC2; AEC1	Mice post PNX	Only Hopx^+^Igfbp2^–^ AEC1 generate AEC2 and AEC1 *in vitro* or in mice under PNX.	Jain et al., 2015; Wang et al., 2018
α6β4^+^ cells	α6^+^β4^+^SPC^–^	Alveoli; Airway	CC10^+^ cells; AEC2; Krt5^+^ cells	Human; bleomycin-injured mice	In bleomycin-injured mice, α6β4^+^ cells quickly expand and express mature epithelial cell markers like CC10 and SPC. Human α6β4^+^ cells can differentiate into Krt5^+^ cells and CC10^+^ cells *in vitro*.	McQualter et al., 2010; Chapman et al., 2011; Li et al., 2013
DASCs	Krt5^+^p63^+^	Distal airway	Club cells; AEC1; AEC2	Human; influenza-, bleomycin-injured mice	DASCs can regenerate airway or alveolar epithelium after severe injury by quick expansion and generation of conducting airway or alveolar epithelial cells.	Kumar et al., 2011; Zuo et al., 2015; Shi et al., 2019
LNEPs	CC10^–^β4^+^ CD200^+^CD14^+^	Distal airway	ΔNp63^+^Krt5^+^ cells; Club cells; AEC1; AEC2	Influenza-, bleomycin-injured mice	LNEPs activate a ΔNp63 and Krt5 remodeling program after influenza infection or bleomycin injury in mice. Transplanted LNEPs can expand and differentiate into mature epithelial cells in the injured lung.	Vaughan et al., 2015
Sox2^+^ progenitors	Sox2^+^H2-K1^high^p63^–^	Distal airway	AEC1; AEC2	Bleomycin-injured mice	After mice were injured by bleomycin instillation, quiescent H2-K1 high cells can expand, migrate, and differentiate into AEC1 and AEC2 to rebuild the denuded alveolar epithelium.	Kathiriya et al., 2020
Sox9^+^ progenitors	Sox9^+^Krt5^+^ p63^+^	Conducting airway	Conducting airway epithelial cells; AEC1; AEC2	Human; bleomycin- or naphthalene-injured mice	Transplanted Sox9^+^ progenitors can differentiate into various conducting or alveolar epithelial cells in injured mice. Engrafted human Sox9^+^ basal cells can improve lung function of bleomycin mice or patients with bronchiectasis.	Nichane et al., 2017; Ma et al., 2018
BASCs	CC10^+^SPC^+^	BADJ	AEC1; AEC2; Club cells; Ciliated cells	Influenza-, bleomycin- or naphthalene-injured mice	BASCs can differentiate into AEC2 and club cells upon influenza virus infection, AEC1 and AEC2 upon bleomycin injury, and club cells and ciliated cells upon naphthalene injury.	Tropea et al., 2012; Liu et al., 2019; Salwig et al., 2019; Liu et al., 2020
v-CCs	CC10^+^Upk3a^+^	BADJ; distal airway	AEC1; AEC2; Club cells; Ciliated cells	Bleomycin- or naphthalene-injured mice	v-CCs have greater potency to differentiate into ciliated cells and club cells during homeostasis or naphthalene injury, and differentiate into AEC1 and AEC2 after bleomycin-induced lung injury.	Giangreco et al., 2002, 2009; Guha et al., 2017

*α6, integrin α6; AEC1 and AEC2, type 1 and type 2 alveolar epithelial cells; β4, integrin β4; BADJ, bronchioalveolar-duct junction; BASCs, bronchioalveolar stem cells; CC10, club cell 10-kDa protein; DASCs, distal airway stem/progenitor cells; ΔNp63, a p63 splice variant; Hopx, HOP homeobox; HTII-280, a 280- to 300-kDa protein specific to human type II cells; Igfbp2, insulin-like growth factor- binding protein 2; Krt5, keratin 5; LNEP, lineage-negative epithelial progenitors; PNX, pneumonectomy; SPC, surfactant protein C; Sox, sex determining region Y (SRY)-box; v-CCs, variant club cells; Upk3a, uroplakin 3a.*

AEC1 have long been considered as terminally differentiated cells, while some studies surprisingly found that they had the plasticity to dedifferentiate into AEC2 (Jain et al., 2015; Wang et al., 2018). Jain et al. (2015) revealed that in mice under partial pneumonectomy (PNX), AEC1 could give rise to AEC1 and AEC2. Wang et al. (2018) further demonstrated that only the Igfbp2-negative AEC1 had such proliferation and differentiation ability. In alveoli or bronchioles of adult mouse, there is a group of multipotent progenitor cells, expressing integrin α6 (CD49f) and β4 (CD104), but negative for SPC (McQualter et al., 2010; Chapman et al., 2011). Upon bleomycin injury, α6β4^+^ cells quickly expand and express mature epithelial cell markers like CC10 and SPC (Chapman et al., 2011). α6β4^+^ epithelial cells also can be isolated from human distal lung but fail to express SPC during *in vitro* culture (Li et al., 2013). Probably certain factors are required to induce their specific transition.

Previous histological data have presented the existence of p63^+^ epithelial cells lining the honeycomb cyst of IPF (Chilosi et al., 2002). Vaughan et al. (2015) discovered a small proportion of Krt5-lineage labeled cells in the injured mouse alveolar epithelium, indicating that the preexisting Krt5^+^ cells from bronchioles might migrate to the alveolar region during wound repair. Several studies have identified distal airway stem/progenitor cells referred to as DASCs, which expressed p63 and Krt5, and could regenerate the bronchiolar and alveolar epithelium after influenza or bleomycin injury in mice (Kumar et al., 2011; Zuo et al., 2015; Shi et al., 2019). Other than the preexisting p63^+^/Krt5^+^ cells, there is a rare subpopulation of progenitor cells in mouse distal airway named as lineage-negative epithelial stem/progenitors (LNEPs) marked by CC10^–^β4^+^CD200^+^CD14^+^ (Vaughan et al., 2015). Quiescent LNEPs are ΔNp63-low or negative and very scarce in normal lung. Upon influenza- or bleomycin- induced severe damage, they can activate a ΔNp63 and Krt5 remodeling program, then expand and migrate widely to repair the injured area. One study identified a subpopulation of Sox2^+^ progenitors with high expression of MHC class I marker H2-K1 (Kathiriya et al., 2020). In bleomycin-injured mice, quiescent H2-K1^high^ cells can expand, migrate and differentiate into AECs. Even when the resident H2-K1^high^ cells are damaged, transplanted H2-K1^high^ cells can survive and function efficiently in the pathological environment of injured lung. Sox9^+^ cells isolated from mouse embryonic lung have multipotency to generate various conducting airway epithelial cells and AECs after transplanted into lung-injured mice (Nichane et al., 2017). Ma et al. (2018) found that xeno-transplanted human Sox9^+^ basal cells could differentiate into AEC1 and ameliorate lung fibrosis of bleomycin-injured mice. And autologous engrafted Sox9^+^ basal cells also improved the lung function of patients with bronchiectasis (Ma et al., 2018).

Bronchioalveolar-duct junction (BADJ) is an intermediate zone connecting the bronchioles and alveoli. Bronchioalveolar stem cells (BASCs) located at BADJ co-express markers of club cells (CC10) as well as AEC2 (SPC) (Tropea et al., 2012; Liu et al., 2019, 2020; Salwig et al., 2019). They tend to generate conducting airway epithelia, AECs or both, after naphthalene- or bleomycin-induced injury or influenza virus infection, respectively. Another progenitor subtype found to exist at BADJ is variant club cell (v-CC), marked by Uroplakin3a (Upk3a) (Giangreco et al., 2002, 2009; Guha et al., 2017). This rare subgroup of club cells also distributes in terminal bronchiolar airway and prefers to gather around neuroepithelial bodies (NEBs), which provide a microenvironment facilitating the development of v-CCs. Similar to BASCs, v-CCs contribute to the bronchiolar epithelium homeostasis with their great potency to generate club cells and ciliated cells, as well as reconstitute alveolar or bronchiolar epithelia during injury (Guha et al., 2017). Migration and transdifferention into AECs of these candidate progenitor/stem cells after lung injury may explain why lung fibrosis would spontaneously resolve in some fibrotic animal models even with AEC2 dysfunction or ablation.

Although animal studies have suggested that non-AEC2 progenitor/stem cells could help resolve lung fibrosis, their exact role in IPF is still unknown. Histologically, alveolar epithelia are denudated and replaced with hyperplastic AEC2 and conducting airway-like cells, including basal cells, club cells and goblet cells (Kawanami et al., 1982; Chilosi et al., 2002; Xu et al., 2016; Prasse et al., 2019). The ectopic existence of airway-like cells in the alveolar epithelium is called “bronchiolization,” representing aberrant alveolar regeneration in IPF lungs. The origin of these airway-like cells has raised great concern. According to single-cell sequencing analysis, most of alveolar cells from IPF patients are both positive for the AEC2 marker (HTII-280) as well as the conduct airway cell marker (such as P63, KRT5, or MUC5AC), indicating that they are at an intermediate state between alveolar cells and airway cells (Xu et al., 2016). Some researchers have supposed that these airway-like cells might originated from the airway, as P63 and KRT5 usually mark airway basal cells and are absent in alveolar progenitor/stem cells. Although animal studies showed that basal cells from large airway can hardly migrate to the alveolar regions (Vaughan et al., 2015; Zuo et al., 2015), it’s still possible that these cells are derived from DASCs (Kumar et al., 2011) or Sox9^+^ basal cells (Ma et al., 2018), as both of them have been found in human lungs and generate mature AECs. Therefore, it’s very likely that non-AEC2 progenitor/stem cells participate in alveolar regeneration during progression of IPF. However, there might be something wrong in this process, leading to abnormal alveolar repair.

## New Perspectives on the Mechanism of Abnormal Alveolar Regeneration in IPF

**FIGURE 1:**
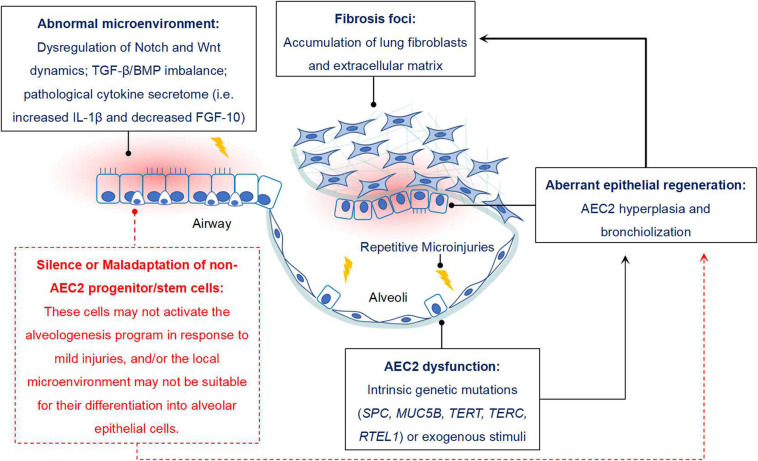
Potential role of non-AEC2 progenitor/stem cells in IPF. Some progenitor/stem cells from distal airway have potential potency of alveologenesis after injury, while they do not function properly in IPF. The reason may be that these cells do not respond to mild lung injury and initiate the program of differentiating into mature AECs. In addition, the resident microenvironment may not be appropriate for their activation, expansion and differentiation. For instance, disregulation of Notch and Wnt signaling dynamics, TGF-β/BMP signaling imbalance, sustained IL-β1 expression and reduced FGF-10 would not favor the differentiation of lung progenitor cells. In combined with the “quiescent state” of other AEC precursors, dysfunction of AEC2 would finally contribute to the aberrant alveolar epithelium regeneration in IPF, and then facilitate the progression of lung fibrosis.

Increasing evidences have suggested that some non-AEC2 progenitor/stem cells, including BASCs, v-CCs, LNEPs, DASCs, H2-K1^high^ progenitors in mouse, and DASCs, Sox9^+^ basal cells in human, might serve as alternate stem cells since they have sufficient capability of alveolar epithelium restoration when AEC2 are dramatically depleted or loss of function. If they work, the lung fibrosis would gradually vanish or even not happen. However, the real condition is not like that. Thus, we propose that during the pathogenesis of IPF, not only AEC2 are impaired, but other epithelial progenitor/stem cells may also be dysfunctional and/or maladaptive (Figure 1).

### Non-AEC2 Progenitor/Stem Cells May Not Activate Upon Mild Injuries

Currently, the potency of the non-AEC2 progenitor/stem cells to restore alveoli is almost only reported in mice under bleomycin treatment and/or influenza infection, which actually induce acute and severe lung injury. It seems that their ability to generate AECs is strictly conditioned. Vaughan et al. (2015) demonstrated that LNEPs were only activated in response to severe lung injury, rather than mild/moderate damage. Similarly, Krt5^+^ stem cells almost only exist in those severely damaged lung regions, while alveolar epithelial progenitors are more likely present in mild/medium injury areas (Zacharias et al., 2018). Accordingly, the level of injury may be an important parameter affecting the activation of non-AEC2 progenitor/stem cells. When alveoli was mildly injured, the majority of activated progenitors were resident AEC2; when alveolar epithelia were severely damaged, the major committed cells might be distal airway- or BADJ-derived progenitor/stem cells, such as LNEPs, BASCs, and DASCs.

IPF is thought to be initiated by chronic multi-microinjuries. However, most of conventional animal models of lung fibrosis are induced by a severe damage. For instance, intratracheally instillation of bleomycin will lead to an acute lung injury response or even death during the early stage (Adamson and Bowden, 1974), while IPF often has an insidious onset and does not have such a phase of acute lung injury before lung fibrosis. In this animal model of lung fibrosis, many non-AEC2 progenitor/stem cells have been found to be activated and help restore the impaired alveolar epithelium (Chapman et al., 2011; Vaughan et al., 2015; Guha et al., 2017; Salwig et al., 2019; Shi et al., 2019). This may account for the self-resolution of bleomycin-induced lung fibrosis. Thus in this regard, bleomycin-induced lung fibrosis does not accurately model the initiation and pathological change of IPF. On the contrary, some recent animal models have successfully simulated the progression of IPF. Instead of chemical stimuli, these animal models received chronic and mild stimuli by genetic or surgical methods, such as specifically inducing cellular senescence in AEC2 by selective depletion of *Sin3a* or telomere repeat binding factor 1 (*TRF1*) (Naikawadi et al., 2016; Yao et al., 2019), or producing an elevated mechanical pressure to the right lung via left pneumonectomy (PNX) (Wu et al., 2020). Under such a moderate injury, the mice surprisingly develop a progressive lung fibrosis rather than a transient one. Thus we hypothesize that under a chronic and mild injury or stimulus, the non-AEC2 progenitor/stem cells may not activate the program of differentiation into AECs. This hypothesis needs to be proved by further investigations, especially the state of non-AEC2 progenitor/stem cells upon the microinjuries or in aged subjects.

### Non-AEC2 Progenitor/Stem Cells May Not Adapt the Abnormal Microenvironment of IPF

Another assumption for the non-feasance of these non-AEC2 progenitor/stem cells is that they may be maladaptive to the abnormal microenvironment in the pathological areas. The biological behaviors of epithelial progenitor/stem cells are regulated by some developmental pathways. Activation of TGF-β, Wnt and Notch pathways is beneficial for the expansion of lung progenitor cells during development or after lung injury. Whereas differentiation of progenitor cells into mature lung epithelial cells requires following deactivation of these pathways (Frank et al., 2016; Finn et al., 2019; Riemondy et al., 2019). Notch signaling takes command of the differentiation of lung progenitors during development (Kiyokawa and Morimoto, 2020). Notch on leads to differentiation into club cells, while Notch off promotes their differentiation into ciliated cells and neuroendocrine epithelial cells. Notch overexpression will induce goblet cell-differentiation but inhibit AEC2-differentiation. For example, high Notch activity facilitates the activation and expansion of LNEPs, while differentiation into mature AECs requires the subsequent loss of Notch. However, in the honeycomb cysts of IPF and scleroderma lungs, canonical Notch signaling target HES1 is upregulated (Vaughan et al., 2015), indicating that the persistent activity of Notch signaling may result in malfunction of LNEPs and account for abnormal alveolar repair in interstitial lung diseases. Similarly, Wnt/β-catenin signaling is also involved in transdifferentiation of distal lung epithelial progenitors into AECs. Wnt activation may direct differentiation of non-AEC2 toward AECs. However, sustained high expression of Wnt may impair the proliferation of nascent AECs (Hu et al., 2020). This indicates that transdifferentiation of uncommitted progenitor cells into AECs may require complicated regulation of signaling dynamics. Secretome in the microenvironment also influences the behaviors of lung progenitor cells. For instance, FGF-10 is essential for the survival and proliferation of epithelial stem cell like DASCs (Zuo et al., 2015; Shi et al., 2019), and it can also promote alveologenesis after lung injury (Yuan et al., 2019), while the level of FGF-10 is declined in progressive IPF patients (Chanda et al., 2016). A recent study showed that treatment with IPF-relevant cytokine cocktail (including IL-1β, TGF-β1, TNF-α, IL-8, IL-33, IL-13, IL-4, TSLP, and MCP-1) altered the differentiation direction of alveolar and small airway epithelial stem cells (Schruf et al., 2020). These evidences indicate that the lower engagement of non-AEC2 progenitor/stem cells in alveolar regeneration may also attribute to the unfavorable microenvironment in IPF lungs.

## Discussion

IPF is a progressive fibrotic disorder driven by the vicious cycle of abnormal epithelial injury/repair. AEC2 are the prime stem cells in alveolar region during hemostasis and lung injury, their dysfunction is considered to play a leading role in the pathogenesis of IPF. However, the contribution of other types of lung progenitor/stem cells in IPF is poorly understood. Some animal studies have implicated that even when AEC2 were specifically depleted, lung fibrosis would still self-resolve, indicating that there might be other progenitor/stem cells getting involved in the alveolar regeneration. In this regard, increasing studies identified numerous non-AEC2 progenitor/stem cells with great plasticity to generate alveolar epithelial cells. Thus, it’s highly possible that these cells may serve as alternate stem cells for restoration of alveolar epithelia when AEC2 are dysfunctional or severely depleted. However, it seems that these stem cells did not perform effectually in IPF lungs, suggesting that AEC2 dysfunction may not be the only mechanism for the disease pathogenesis. In IPF, the function of non-AEC2 stem cells may also be in disorder.

According to the current evidences, we come up with hypotheses on why the non-AEC2 stem cells in IPF lungs do not exercise their ability to reconstitute alveolar epithelia. The first possibility is that these cells may only respond to acute and severe lung injury, such as bleomycin instillation or influenza infection. Thus in condition of chronic and mild injuries, these cells are unlikely to be activated or to initiate an alveologenesis program. Another explanation is that the progenitor/stem cells may not adapt to the pathological microenvironment in IPF lungs. The aberrant repair of alveolar epithelia in IPF may be explained by one of these two mechanisms, or the combination of them. Since current evidences are limited, we need further investigations to prove these ideas, especially the state of non-AEC2 progenitor/stem cells in IPF. Studies like this would help illustrate a more concrete process of lung fibrosis, such as explain the existence of “bronchiolization” in honeycomb cysts, and also provide new insights for the treatment of IPF.

## Author Contributions

XT conceived and designed the manuscript, provided guidance, and edited the manuscript. Both authors wrote the manuscript and critically revised it.

## Conflict of Interest

The authors declare that the research was conducted in the absence of any commercial or financial relationships that could be construed as a potential conflict of interest.

## Abbreviations

α 6: integrin α 6
AEC1: type I alveolar epithelial cells
AEC2: type II alveolar epithelial cells
β 4: integrin β 4
BADJ: bronchioalveolar-duct junction
BASCs: bronchioalveolar stem cells
BMP: bone morphogenetic protein
CC10: club cell 10-kDa protein
DASCs: distal airway stem/progenitor cells
Δ Np63: a p63 splice variant
ER: endoplasmic reticulum
FDA: food and drug administration
FGF-10: fibroblast growth factor-10
HES1: hairy and enhancer of split 1
Hopx: HOP homeobox
HTII-280: a 280- to 300-kDa protein specific to human type II cells
Igfbp2: insulin-like growth factor- binding protein 2
IPF: idiopathic pulmonary fibrosis
Krt5: keratin 5
LNEPs: lineage-negative epithelial stem/progenitor
PNX: pneumonectomy
RTEL1: regulator of telomere elongation helicase 1
SA β-gal: Senescence-associated β-galactosidase
Sox: sex determining region Y (SRY)-box
SPC: surfactant protein C
TERC: telomerase RNA component
TERT: telomerase reverse transcriptase
TRF1: telomere repeat binding factor 1
UIP: usual interstitial pneumonia
Upk3a: uroplakin 3a
v-CCs: variant club cells.
